# The gastrointestinal and microbiome impact of a resistant starch blend from potato, banana, and apple fibers: A randomized clinical trial using smart caps

**DOI:** 10.3389/fnut.2022.987216

**Published:** 2022-09-29

**Authors:** Douglas Hanes, Brent Nowinski, Joseph J. Lamb, Ilona A. Larson, Daniel McDonald, Rob Knight, Se Jin Song, Noelle Patno

**Affiliations:** ^1^National University of Natural Medicine, Helfgott Research Institute, Portland, OR, United States; ^2^Center for Microbiome Innovation, University of California, San Diego, San Diego, CA, United States; ^3^Personalized Lifestyle Medicine Center, Gig Harbor, WA, United States; ^4^Formerly Metagenics, Inc., Aliso Viejo, CA, United States; ^5^Department of Pediatrics, University of California, San Diego, San Diego, CA, United States; ^6^Department of Bioengineering, University of California, San Diego, San Diego, CA, United States; ^7^Department of Computer Science and Engineering, University of California, San Diego, San Diego, CA, United States

**Keywords:** prebiotic, monitoring, microbiome, *Faecalibacterium*, *Akkermansia*, compliance, fiber, sleep

## Abstract

The gastrointestinal (GI) impact of fibers including resistant starch (RS) consumption depends on various types and amounts of fibers, the initial microbiome states, and accurate intake measurements. A randomized clinical trial evaluated the GI impact of varying doses of a novel resistant starch blend (RSB) with smart cap monitoring. RSB contained at least 50% RS and was a proprietary mixture of a potato starch, green banana flour, and apple fiber powder (a source of apple pectin, not resistant starch). The study design randomized participants to one of four arms: 10 g/day of potato starch (0 RSB), 10 g/day of RSB, 10 to 20 to 20 g/day of RSB or 10 to 20 to 30 g/day RSB for two-week intervals over 6 weeks. Results confirmed that while resistant starch of approximately 5 g per day improves GI symptoms at 2, 4, and 6 weeks, it did not demonstrate a detectable effect on short chain fatty acids. Increasing doses of the blend (RSB) led to a decrease in the diarrhea score. Using an estimate of total consumption of RSB based on smart cap recordings of container openings and protocol-specified doses of RSB, a reduction in the sleep disturbance score was associated with higher RSB dose. The exploratory microbiome evaluation demonstrated that among the 16S rRNA gene sequences most associated with the consumption of the novel blend RSB, two belong to taxa of notable interest to human health: *Faecalibacterium* and *Akkermansia*.

## Introduction

The recommended dose of total dietary fiber is currently set at 25–38 g/day in the US depending on the person's caloric needs, based on a 14 g/1,000 kcal adequate intake (1). This established intake is well-supported by epidemiological studies identifying the cardiovascular disease prevention benefit, with additional evidence for reducing risk of developing type II diabetes and colon cancer, improving gastrointestinal health and body weight control, and lowering risk of mortality (1–4). However, there is not a universally recommended daily dietary amount of resistant starch, nor is there an understanding of which sources of fiber or resistant starch need to comprise the 25–38 g/day (1). Most dietary fiber and resistant starch trials show benefits for a single ingredient above 15 g/day (1, 5, 6) yet it is unknown whether a blend of resistant starch and fibers may provide equivalent or improved benefits.

Of the four types of resistant starch, typically the retrograded starch (type III, example: cooked and cooled potatoes) and type II are the most common in the diet. Resistant starch type II, which is starch that escapes digestion in the small intestine due to its natural granular structure, can be found in high-amylose maize starch (HAMS), green banana starch, and raw potato starch (4). Resistant starch contains amylase-resistant glycans, resists digestion in the upper GI tract and has been shown to be metabolized by colonic amylolytic bacteria such as *Ruminococcus bromii* (7). It shows promise for controlling blood glucose and insulin levels, as well as acting as a prebiotic (“a substrate that is selectively utilized by host microorganisms conferring a health benefit”) (8) by modulating the microbiome (4, 9). However, studies suggest that effects may be dependent on both dose and type of RS content (10–12).

High levels of resistant starch have been associated with health benefits and altering gut microbiota levels. The most recognized RS research supports the glucose-lowering benefit for reducing the risk for type 2 diabetes and used 15–40 g/day of HAMS (13–15). At the quantity of 159 g/day, HAMS (containing 66 g of RS) altered endogenous microbiota levels, including increasing the beneficial *Faecalibacterium* (10). Relative to stool samples from healthy individuals, *F. prausnitzii* has been found at lower levels in diseases including inflammatory bowel diseases (particularly Crohn's), irritable bowel syndrome, colorectal cancer (16), severity of coronavirus disease (COVID-19) (17) and cystic fibrosis (18). It has been shown to exhibit anti-inflammatory effects through butyrate production and immune cell modulation (19). As an obligate anaerobe, difficult to cultivate and not yet developed as a probiotic, *F. prausnitzii* has been a next-generation microbe of interest to target with prebiotics (20).

Interestingly, *F. prausnitzii* has also been found to be associated with individuals who consume a high diversity of plants in their diet (21), suggesting that this species may be influenced by components besides resistant starch. Consistent with this idea, *in vitro* studies have shown that specifically apple pectin (a fiber but not a resistant starch) supports the growth of multiple strains of *F. prausnitzii* (22, 23). Pectin also has greater specificity in stimulating specific microbes' growth and short-chain fatty acids when compared to fructooligosaccharides (FOS) and resistant starch type II (24). Extrapolating from these *in vitro* studies, apple pectin has the potential to modulate *F. prausnitzii* at lower doses than the quantities observed from RS intake studies.

Raw potatoes and green bananas provide alternative sources for resistant starch and also have been studied for glucose control benefits and microbiome impact. While the concentration in HAMS is 46%, raw potato starch contains a higher quantity (63%) and green banana flour contains similar quantities (44%), as described in the Association of Official Analytical Collaboration (AOAC) 2002.02 method publication (25). Although native potato starch has the highest content, its consumption at 48 g/day yielded variable responses in butyrate production which may have been due to differences in the initial microbiomes of the young men (26). Studies using 30 g raw potato starch/day for 12 weeks led to relative increases in *bifidobacteria* and improved glycemic responses in elderly people (27, 28). In other studies, 38.3–40 g of native banana starch improved postprandial glycemic responses and reduced supplemented meal consumption (29, 30). A few studies have shown daily consumption of various amounts and types of resistant starch (17–66 g) resulted in higher levels of SCFA (10, 12, 26, 31–33) in as little as 1–3 weeks. Yet it remains to be explored whether a diversity of fibers at a lower dose may obtain GI and glycemic benefits in a short period of time or whether the baseline microbiome or other characteristics impact a person's response to such an intervention.

Disparities in study outcomes may also be due to differences in participant consumption. Adherence to protocols can vary for reasons including anticipated negative effects of consuming large amounts, such as flatulence resulting from 39 g (12). Moreover, traditional methods of self-reporting can overestimate adherence by 17%, and pill count can overestimate by as much as 8%, which suggests that alternative methods may need to be developed to track consumption more accurately, such as electronic detection of package opening by the participant (34). Measuring methods such as tracking in a log, performing a pill count, or weighing the supplement remaining after being dispensed have been utilized, and adherence in a clinical trial has been observed to be as low as 46 or 55% (35). For this study, a unique smart cap designed to detect acceleration of the cap rotation, and subsequent flipping upside-down (36) was used to track opening of the supplement container.

The resistant starch blend of natural fibers was developed to deliver a high quantity of resistant starch (utilizing raw potato starch), to utilize a diversity of resistant starches (with the addition of green banana flour) and to deliver a positive impact to the microbiome (e.g., anticipated to be enhanced with the addition of apple pectin). The primary aim was to evaluate the impact compared to a single-source RS from potato on short-chain fatty acids (SCFA), specifically butyrate. The study was designed to evaluate doses of the blend ranging between 10–30 g/day in 2–6 weeks for its impact on SCFA production, GI symptoms, well-being, and sleep measures, as well as explore the impact on the gut microbiome. With a diversity of ingredients, the increasing doses of the resistant starch blend (RSB) was anticipated to increase SCFA, improve GI symptoms, and possibly alter microbiota in ways associated with human health.

## Materials and methods

### Eligibility

The clinical trial ran from June 2019 through December 2020 at the Personalized Lifestyle Medicine Center (PLMC) in Gig Harbor, WA as a single-center, randomized, blinded, placebo-controlled parallel trial. Men and women ages 21–65 years, self-reporting on the presence of minor bloating, constipation, or irregular bowel movements, were recruited. Exclusion criteria were the following: unwillingness to follow study procedures; current or recent consumption of probiotics, resistant starch, prebiotic, or fiber supplements (14 days before first stool collection); current or recent (within last 28 days) use of antibiotic, antiparasitic, or antifungal drugs; current use of supplements or medications such as proton pump inhibitors (PPIs), opioids, or selective serotonin reuptake inhibitors (SSRIs) that may impact GI motility; current unstable or serious illnesses or infections or cardiovascular diseases; history of diabetes or hypoglycemia or prediabetes; personal history of mental illnesses; known allergy or intolerance to supplement ingredients; diseases affecting digestion and absorption of nutrients; GI/bariatric surgery within the last 5 years; current colostomy/ileostomy; genitourinary bacterial infections within the last 28 days; major hospitalizations within the preceding 3 months; skin or cervical cancer within the last 5 years; current or recent (past 30 days) use of nicotine or smoking; alcoholism or diagnosis within the last 12 months and during study; alcohol consumption that was more than 2 glasses at a time or more than 4 glasses within the prior two weeks; use of recreational drugs within 12 months prior and during the study; major changes to diet or exercise within 28 days of screening or during study, including dietary weight loss program; and current or recent (within 28 days) involvement in another interventional study. To minimize impact to the intestinal microbiota, alcohol usage was limited to 1–2 glasses of light beer or wine for 1–2 days after stool collection during the study for those who selected Track A, while the majority selected Track B, the no-alcohol consumption option during the study. Participants were primarily recruited locally via flyer and word of mouth, online via the Personalized Lifestyle Medicine Center's Facebook page, and then expanded to other states through multiple online ads during the COVID-19 pandemic-associated lockdown when the trial became a virtually conducted, remote study.

The study protocol was approved by Aspire Institutional Review Board (IRB) on 14 May 2019. The IRB tracking number is 520190117. All participants provided written informed consent. Trial registration number at ClinicalTrials.gov: NCT03983772.

### Study design and randomization

The study design included four groups with one on a potato starch for 10 g a day for 6 weeks and three groups on RSB in a dose-ramping design as follows: group 1 on 10 g/day of RSB for 6 weeks, group 2 taking 10 g/day of RSB for 2 weeks and then increasing the dose from 10 g/day to 20 g/day of RSB for the next 4 weeks, and group 3 taking 10 g/day of RSB for 5 weeks, then 20 g/day of RSB for 2 weeks, and finally 30 g/day of RSB for 2 weeks (see Supplementary Figure S1). Participants were advised to consume their RSB cold or cool, mixed in liquid or food, and were asked to track how they took the RSB. All groups experienced a 2-week run-in period prior to the interventional supplement consumption for baseline measurements to evaluate physiological variability of the measured outcomes. Power calculations used data from previously published work from Phillips et al. (12), a randomized crossover design testing 5 v. 39 g/day resistant starch consumption that resulted in 7.2 mmol/L more butyrate following the high RS consumption after 3 weeks. We calculated that, with 10 participants per group, we would have 80% power to detect a mean change of 7.2 mmol/L in butyrate between two independent groups using an independent *t*-test design (calculations made using STATA v.14). The intended enrollment number was therefore determined to be 40 with 10 per group. PLMC study staff randomly assigned participants in a 1:1:1:1 ratio by using the program at https://www.randomizer.org to associate group assignments with the order of enrollment. Participants were randomized after they passed screening but prior to receiving the supplement. For any participants who withdrew prior to receiving supplement, corresponding group assignments were re-entered at the end of the randomization list for reassignment to new subjects entering the study. Participants and physician were blinded to the product assignment at the beginning of the study. For a few adverse events, the physician and participant, upon request of the study staff, were informed of the product assignment.

All participants were asked to maintain their current diet, exercise, and lifestyle habits and were provided with a list of foods to avoid and a list of foods to maintain similar consumption throughout the study (to minimize changes in the possible confounder of dietary fiber intake throughout the study) (list included in Supplementary Table S1). To monitor dietary consumption throughout the study, a 24-h food recall was requested within 3 days of each visit. Participants were asked to provide the dietary recall immediately after stool collection as much as possible. Dietary intake data for 24-h recalls were collected and analyzed using the Automated Self-Administered 24-h (ASA24) Dietary Assessment Tool, version 2018 developed by the National Cancer Institute, Bethesda, MD (37). The data extracted from the ASA24 reports for subsequent analysis were total calories (kilocalories), fat (as a percentage of kilocalories), protein (in grams), carbohydrates (in grams), and fiber (in grams). The fiber intake reported on the ASA24 did not include the fiber in the intervention, as consumption of the intervention was recorded on the product dosing log, extrapolated from the product weight and the smart cap monitoring.

### Material and smart caps

The material for the intervention was a proprietary RSB containing the clinically-studied (27, 28) native potato starch (MSPrebiotic^®^ Carberry, Manitoba, Canada), green banana flour (Nubana; Alsip, IL) and a source of apple pectin from apple fiber powder (Mayer Brothers; West Seneca, NY). The resistant starch content of the RSB was designed to be at least 50% per the AOAC 2002.02 method and was confirmed in prior batches. The comparison was a native potato starch called Potato Starch Superior from Emsland (Piscataway, NJ) referenced herein as PS for Potato Starch). The lot used for quality acceptance testing of the Emsland potato starch material resulted in < 5% of resistant starch per the AOAC 2002.02 method. While the supplier's product specification stated that only traces of fiber were present, test results of product returned by participants confirmed amounts of overall RS equivalent to that in the RSB; RSB resistant starch content was 55.7% and the potato starch resistant starch content was 52.6% (AOAC 2002.02 method, Covance lab, Madison, WI). Repeating the testing on the potato starch resulted in 68.4% for the resistant starch, and 72.0% for the total fiber content, using the 2011.25 method. The RSB test results for fiber were 55.8% soluble fiber with 12.3% insoluble fiber while the potato starch had 59.0% soluble fiber and 12.9% insoluble fiber (AOAC 2009.01 and 2011.25 methods, Covance Lab, Madison, WI). Metagenics, Gig Harbor, WA, tested the RSB and Emsland Potato Starch for heavy metals and microbiological contamination prior to releasing the clinical test product for use. Material was packaged in jars with “smart” caps to track intake compliance using an internal monitor to detect each event that the cap had been twisted off and set upside down with a time and date stamp (US Patent 10,874,591 B2) (36). Smart caps were generously provided by Amway (Ada, MI).

### Outcomes

The primary outcomes of the study were fecal butyrate, total SCFA, acetate, and propionate, as obtained from self-collected samples. The protocol requested stool sample collection to occur within 3 days of the protocol-scheduled visit based on the participant's ability and motility (see Supplementary Figure S1). Participants used the pink-top tube which contained preservative from the Genova Diagnostics' test kit, which includes a built-in scoop in the tube lid for participants to collect stool from the stool deposited into a container. Participants shipped tubes per the kit manufacturer's instructions (Federal Express) to Genova Diagnostics for analysis. Fecal SCFA test results were provided by Genova Diagnostics (Asheville, NC) using gas chromatography-mass spectrometry (GC-MS) as described by Lihong et al. (38).

Secondary outcomes included the Bristol Stool Form (Bristol Stool chart rating) and fecal frequency (using the average across 7 consecutive days reported within each two-week interval between visits) as well as GI symptoms from validated questionnaires (39). Patient Reported Outcomes Measurement Information Systems (PROMIS) Scale v1.0—GI Diarrhea 6a T-score, PROMIS Scale v1.0—GI Constipation T-score, and PROMIS Scale v1.0—GI Gas and Bloating 13a T-score collected at each visit. T-scores are also simply called PROMIS scores in this manuscript.

Exploratory outcomes included wellbeing as assessed by validated questionnaire Quality of Life in Neurological Disorders (Neuro-QoL) Item Bank v1.0—Positive Affect and Well-Being (40), PROMIS Short Form v1.0—Sleep Disturbance 8b (41), standard lipid panel (total cholesterol, LDL, triglycerides, and HDL); insulin (through the Comprehensive Metabolic Panel from QuestQuanum, Quest Diagnostics, (West Hills, CA and Seattle, WA); and evaluation of microbiome changes through the American Gut Project (DNA sequencing of the V4 region of the 16S rRNA gene) (21).

Fasted blood draws were performed at visits 1, 2, and 5 to evaluate physiological changes at run-in and the 6-week impact of the intervention on cholesterol and insulin as exploratory outcomes, as well as serving as a safety check. These safety measures involved the following: vital signs (blood pressure, respiration rate, pulse), height (first visit only), body weight, and blood draws (previously described). Compliance measures included the following: ASA24 (previously described), a supplement container using a “smart” cap that counted times rotated and turned upside down for container openings (36) and supplement weight measurements and participant logs of supplement consumption.

Vital signs and blood draws were suspended during the COVID-19 pandemic associated lockdown while telehealth rather than in-person visits were utilized.

### Statistical analysis

**Figure 1:**
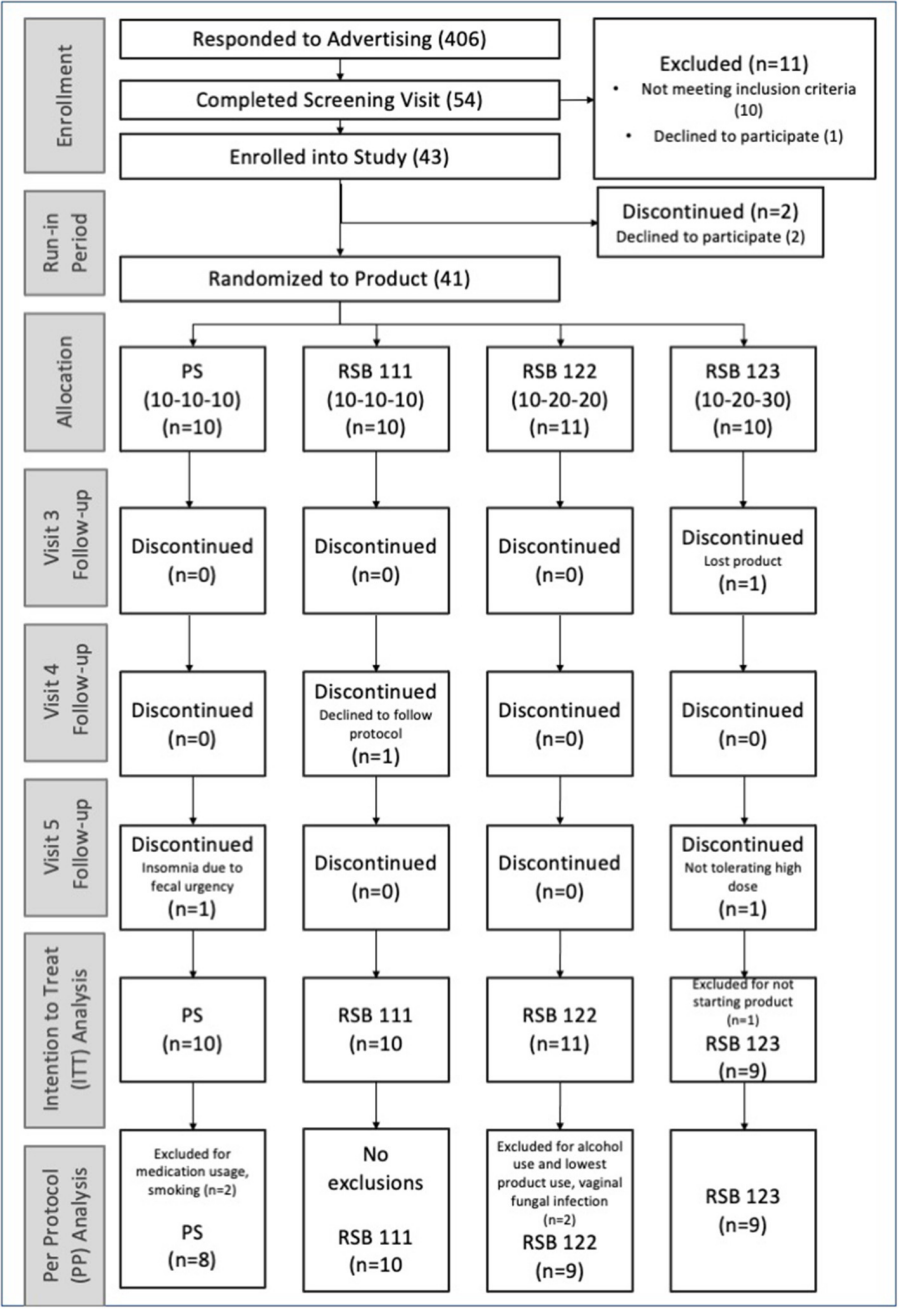
Consort diagram. Enrollment of the participants and numbers per group included in the ITT and PP analyses. Randomization to product occurred at visit 2.

The intention-to-treat (ITT) analysis included all participants enrolled in the study, regardless of adhering to restricted alcohol intake or medication use during the study (refer to Figure 1). An evaluation of the protocol criteria was performed on the data collected in study visits with the physician as well as evaluating the consumption of supplement weight (using a minimum cut-off of 50% for the supplement weight and a minimum of 70% for the dosing log). The per-protocol (PP) analysis excluded four participants due to alcohol consumption prior to stool collection, smoking, excluded medications, an ongoing infection present at baseline; the PP analysis also excluded visit 5 data from two participants who discontinued supplementation (refer to the CONSORT diagram, Figure 1).

For missing data in the fecal SCFA category, we imputed values returned as below the detection level using random imputation between the reported lower detection limit and/or the lowest reported value (whichever was lower) and one-half that value. Data missing for all other outcome measures was not replaced. The PROMIS outcome measures were scored using the HealthMeasures Scoring Service, an online application powered by Assessment CenterSM. Means and confidence intervals were calculated for the primary and secondary outcome measures from both the ITT and PP analyses for each visit as recommended by CONSORT guidelines (42).

For comparison across time points and categories, the baseline was established as an average of the data from visit 1 and visit 2 (or the value at one of these visits, if the other was missing), minimizing data loss. Differences in outcomes between groups were assessed using linear mixed models with a random intercept and either a factor for randomized group or a measure of time-dependent dose as the main predictor. Significant effects of randomized group were followed by pairwise comparisons of treatment dose groups against PS. For smart cap analysis, the percentage of smart cap usage was calculated as the number of days having at least one event of using the smart cap per day during the time period between visits divided by the total number of days during the visits (during which consumption was expected). This smart cap percentage was multiplied by the expected delivered dose of RSB per day to determine the “smart cap dose” and was summed over the 6 weeks for the “total smart cap dose.” The smart cap dose thus reflected the dose of g of RSB and was zero for all those in the PS group. Counting days tracked of supplement consumption using participant logs and using the dispensed supplement weight were alternative measures of estimating supplement consumption also used to explore effects on secondary and exploratory questionnaire outcome measures. Additionally, fiber intake from the ASA24 questionnaires was used to assess as a confounder. Statistical analysis of treatment effects was carried out using R v.4.0.2 (43).

### Microbiome analysis

Stool samples were collected and processed using the American Gut Project protocols for analysis of the V4 region of the 16S rRNA gene (21). Briefly, DNA was extracted using the Qiagen MagAttract PowerSoil kit in three separate batches, the V4 region was amplified using the 515f/806r primer set as described in the Earth Microbiome Project [(http://www.earthmicrobiome.org/protocols-and-standards/16s), and the resulting pool was sequenced on an Illumina MiSeq instrument] in two separate runs. Raw reads were demultiplexed and quality-filtered using Qiita (44) keeping reads with a Phred score of 4 or higher, trimmed to 150 nt, and denoised to amplicon sequence variants (ASVs) using deblur v1.1.0 (45). A phylogenetic tree for diversity analyses was created through fragment insertion with the Greengenes v13_8 as a reference backbone (46, 47). Microbiome analyses with amplicon sequencing variants (ASVs) were run using QIIME2-2020.6 (48). Amplicon sequencing variants (ASVs) were classified using the SILVA 138 release (49), and taxa to bloom during the storage of samples prior to processing were removed as previously described (50). For alpha and beta diversity analyses, ASV tables were rarified to 1,400 sequences per sample, and metrics were calculated using the Q2 core-metrics-phylogenetic plugin. Prior to rarefying, 6 samples with fewer than 1,400 reads were discarded, and the remaining samples ranged from 1,410 to 46,684 reads (average: 19,161). Robust Aitchison PCA (RPCA) was also run on the unrarefied data to verify that results were not influenced by rarefaction. Longitudinal analyses and linear mixed effect models of beta diversity across timepoint were done using the QIIME longitudinal plugin. Ranked differential abundance of taxa associated with RSB vs. PS was done through the Q2 Songbird plugin (51) on the unrarefied data with the following parameters: batch_size = 14, summary_interval = 0.01, epochs = 40,000, num_random_test_examples = 11, and differential_prior = 0.5. Log-ratios of the top and bottom 10% of ranked ASVs associated with RSB or PS were visualized and extracted using Qurro (52), and Kruskal-Wallis rank sum test computed with the kruskal.test function in R version 4.1.1 (53).

## Results

### Characteristics of the participants

Of 406 advertisement respondents, 54 people completed the screening visit; of these, 10 were not eligible due to protocol criteria, and one elected not to participate. Of the remaining 43 enrolled into the study, two withdrew before starting the supplement. Participants were randomized to groups at the second visit, before receiving the supplement. Four participants dropped out after receiving the supplement. Two of these participants were not able to comply with the protocol during the study: one lost the supplement, and the other declined to follow instructions. The other two dropped out due to lack of tolerance: one did not want to take the 30 g of RSB and another stopped the 10 g/day PS. The latter reported to the physician insomnia associated with fecal urgency while taking the potato starch. As planned, 40 participants were enrolled and included in the Intention-To-Treat (ITT) analysis. While 37 participants completed the study, 36 qualified for consideration in the Per Protocol (PP) Analysis (Figure 1). Demographic characteristics are listed in Table 1. Measures of body mass index and vitals were only obtained from a subset of participants prior to the study's becoming a remotely conducted study and therefore not included in the final analysis.

**Table 1:** Baseline characteristics of the study population.

	**PS**	**RSB111**	**RSB122**	**RSB123**	**Entire Cohort**
Age (years, range)	27–50	22–59	24–64	24–59	22–64
Age (years, mean ± SD)	39.4 ± 5.9	45.3 ± 12.5	45.9 ± 14.3	35.2 ± 11.2	41.7 ± 11.9
Sex (number, %)					31
Female	5 (50%)	10 (100%)	11 (100%)	5 (56%)	(77.5%)
Male	5 (50%)	0	0	4 (45%)	9 (22.5%)
Race (number, %)					32 (80%)
White	6 (60%)	8 (80%)	9 (81.8%)	9 (100%)	2 (5%)
Black	1 (10%)	1 (10%)			4 (10%)
Asian, Pacific Islander	2 (20%)	1 (10%)	1 (9.1%)		2 (5%)
N/A – did not disclose	1 10%)		1 (9.1%)		
ethnicity, (number, %)					14 (35%)
Not Hispanic	4 (40%)	3 (30%)	4 (36.4%)	3 (33.3%)	1 (2.5%)
Hispanic			1 (9.1%)		
N/A – did not disclose	6 (60%)	7 (70%)	6 (54.5%)	6 (66.7%)	25 (62.5%)

Data are means ± standard deviation (SD), or number (%).

### The dose effect of the blend (RSB)

Table 2 presents the values of the SCFA and GI symptom outcomes, ITT analysis. Supplementary Table S2 presents the values of the exploratory outcomes for the ITT analysis and Supplementary Table S3 presents the values for all outcomes for the PP analysis.

**Table 2:** Changes in SCFA and GI symptom outcomes during the study, ITT analysis.

**Outcome**	**Group**	**Baseline**	**Visit 3**			**Visit 4**			**Visit 5**		
		**Mean**	**Mean ±SD**	**Mean change (CI)**	***p*-value**	**Mean ±SD**	**Mean change (CI)**	***p*-value**	**Mean ±SD**	**Mean change (CI)**	***p*-value**
Acetate (micromole/g)	PS	26.34 ± 12.64	24.44 ± 16.73	0.03 (−7.04 to 7.09)	0.99	38.65 ± 26.96	7.93 (−12.49 to 28.36)	0.38	35.42 ± 26.66	7.42 (−14.21 to 29.05)	0.44
	RSB111	29.17 ± 11.43	17.76 ± 12.05	−11.41^*^ (−21.82 to −1.01)	0.03	32.00 ± 22.92	4.75 (−12.90 to 22.40)	0.54	19.56 ± 11.95	−6.63 (−13.36 to 0.10)	0.05
	RSB122	26.54 ± 18.3	31.99 ± 15.95	5.45 (−4.25 to 15.16)	0.24	38.03 ± 29.3	13.32 (−2.68 to 29.32)	0.09	29.47 ± 11.96	2.93 (−9.72 to 15.59)	0.62
	RSB123	27.01 ± 14.7	33.96 ± 11.52	4.06 (−3.08 to 11.2)	0.22	40.23 ± 13.74	10.33 (−7.41 to 28.08)	0.21	40.34 ± 12.84	10.44 (−7.09 to 27.98)	0.20
Propionate (micromole/g)	PS	10.70 ± 6.66	7.50 ± 5.54	−2.04 (−4.46 to 0.39)	0.09	9.52 ± 6.36	−1.99 (−5.56 to 1.57)	0.22	11.64 ± 9.53	1.03 (−5.66 to 7.72)	0.73
	RSB111	11.80 ± 6.36	7.89 ± 3.53	−3.90 (−7.98 to 0.17)	0.06	11.19 ± 7.54	−0.03 (−6.01 to 5.95)	0.99	7.86 ± 4.18	−2.54^*^ (−4.49 to −0.59)	0.02
	RSB122	11.37 ± 6.55	10.41 ± 5.01	−0.96 (−3.61 to 1.69)	0.44	13.45 ± 9.72	2.32 (−2.32 to 6.96)	0.28	12.45 ± 9.14	1.08 (−4.15 to 6.32)	0.65
	RSB123	11.93 ± 6.60	13.18 ± 5.01	−0.02 (−5.09 to 5.06)	0.99	15.17 ± 5.49	1.98 (−5.07 to 9.02)	0.53	13.75 ± 4.12	0.56 (−5.96 to 7.07)	0.85
n-Butyrate (micromole/g)	PS	10.97 ± 8.29	10.39 ± 5.77	1.12 (−3.11 to 5.35)	0.55	13.24 ± 8.59	0.34 (−6.05 to 6.72)	0.90	13.58 ± 10.31	1.74 (−4.96 to 8.45)	0.56
	RSB111	10.74 ± 4.37	7.97 ± 5.54	−2.78 (−5.48 to −0.07)	0.05	13.91 ± 8.41	3.64 (−1.91 to 9.19)	0.16	8.30 ± 5.34	−2.28 (−4.61 to 0.06)	0.05
	RSB122	12.48 ± 8.35	11.63 ± 7.35	−0.85 (−3.25 to 1.54)	0.44	12.89 ± 7.94	1.31 (−2.77 to 5.39)	0.48	12.39 ± 8.53	−0.09 (−8.26 to 8.08)	0.98
	RSB123	9.99 ± 3.94	11.25 ± 3.92	0.21 (−3.48 to 3.89)	0.90	15.64 ± 7.54	4.59 (−1.86 to 11.05)	0.14	11.31 ± 4.48	0.27 (−4.25 to 4.79)	0.89
Total SCFA (micromole/g)	PS	47.38 ± 26.88	40.82 ± 28.47	−1.61 (−13.53 to 10.32)	0.76	60.71 ± 41.46	5.91 (−19.40 to 31.23)	0.59	59.98 ± 45.77	9.83 (−24.47 to 44.13)	0.52
	RSB111	51.45 ± 18.95	32.46 ± 19.09	−18.99^*^ (−35.46 to −2.52)	0.03	56.52 ± 38.17	8.03 (−20.37 to 36.43)	0.53	34.61 ± 21.8	−12.27^*^ (−21.75 to −2.79)	0.02
	RSB122	50.14 ± 32.47	54.04 ± 27.02	3.90 (−10.09 to 17.88)	0.55	64.37 ± 46.23	17.25 (−6.93 to 41.43)	0.14	54.19 ± 27.46	4.05 (−20.61 to 28.71)	0.72
	RSB123	48.28 ± 25.45	58.38 ± 19.06	4.24 (−9.17 to 17.65)	0.48	71.05 ± 25.52	16.91 (−12.14 to 45.96)	0.21	65.41 ± 20.34	11.28 (−16.27 to 38.82)	0.37
Diarrhea (PROMIS score)	PS	50.11 ± 7.86	49.72 ± 9.95	−0.39 (−3.99 to 3.21)	0.81	48.18 ± 9.68	−1.93 (−4.47 to 0.60)	0.12	47.27 ± 7.81	−2.84 (−7.63 to 1.95)	0.21
	RSB111	45.70 ± 6.49	42.37 ± 5.23	−3.33 (−7.18 to 0.53)	0.08	43.62 ± 5.12	−1.32 (−4.54 to 1.91)	0.37	45.24 ± 6.21	0.31 (−4.34 to 4.95)	0.88
	RSB122	48.40 ± 8.27	45.32 ± 6.99	−3.08 (−6.10 to −0.06)	0.05	42.15 ± 5.22	−6.25^*^ (−10.62 to −1.89)	0.01	43.40 ± 7.03	−5.00^*^ (−9.77 to −0.23)	0.04
	RSB123	45.10 ± 5.01	43.53 ± 4.48	−1.57 (−4.29 to 1.15)	0.22	44.67 ± 6.63	−0.43 (−3.58 to 2.71)	0.76	41.46 ± 3.85	−3.64^*^ (−7.04 to −0.25)	0.04
Constipation (PROMIS score)	PS	50.99 ± 5.55	47.18 ± 7.54	−3.60 (−10.71 to 3.51)	0.28	46.00 ± 8.44	−4.78 (−9.67 to 0.12)	0.05	48.28 ± 8.71	−2.71 (−8.06 to 2.64)	0.28
	RSB111	52.31 ± 7.94	48.77 ± 7.51	−3.54 (−9.35 to 2.27)	0.20	48.62 ± 7.53	−4.37 (−10.42 to 1.69)	0.13	47.20 ± 9.17	−5.79 (−12.75 to 1.18)	0.09
	RSB122	53.10 ± 8.73	48.45 ± 8.26	−4.65^*^ (−8.51 to −0.79)	0.02	48.10 ± 6.10	−5.00^*^ (−9.49 to −0.50)	0.03	47.12 ± 6.09	−5.98 (−12.16 to 0.20)	0.06
	RSB123	49.30 ± 5.88	42.56 ± 7.05	−6.74^**^ (−10.54 to −2.95)	0.003	44.42 ± 9.52	−4.88 (−10.83 to 1.08)	0.10	43.70 ± 7.71	−5.60^*^ (−10.74 to −0.46)	0.04
Gas and bloating (PROMIS score)	PS	58.10 ± 5.88	53.58 ± 7.94	−4.59^*^ (−7.75 to −1.42)	0.01	50.18 ± 9.74	−7.99^*^ (−13.89 to −2.09)	0.01	51.20 ± 7.86	−6.90^*^ (−12.03 to −1.77)	0.01
	RSB111	59.28 ± 7.65	55.37 ± 8.25	−3.91 (−10.62 to 2.8)	0.22	54.04 ± 6.41	−5.43 (−12.99 to 2.12)	0.14	52.67 ± 8.29	−6.81^*^ (−11.94 to −1.68)	0.02
	RSB122	56.29 ± 5.42	53.46 ± 8.81	−2.83 (−8.11 to 2.45)	0.26	52.05 ± 6.03	−4.24^*^ (−7.71 to −0.77)	0.02	50.54 ± 6.07	−5.75^*^ (−10.56 to −0.95)	0.02
	RSB123	56.34 ± 6.75	52.79 ± 6.52	−3.56 (−7.43 to 0.32)	0.07	51.81 ± 7.04	−4.53^*^ (−7.66 to −1.41)	0.01	51.30 ± 7.76	−5.04 (−11.36 to 1.27)	0.10

Data are means ± SD. Mean of the change (size of effect) in subjects with available data from baseline to the respective visit are presented with the confidence interval (CI). Listed *p*-values are for the respective visit as evaluated against the baseline (average of visit 1 and 2 values) within the group assessed by paired comparison t-tests.

* *p*-value < 0.05;

** *p*-value < 0.01.

Significant effects of the dose of the blend were found for the PROMIS Diarrhea T-Score and Bristol Stool Form Score. Using the time-dependent dose predictor variable in the linear mixed model, higher doses of RSB were associated with reductions in diarrhea when adjusted for baseline in the ITT analysis (*p* = 0.021, *p* = 0.082 in PP analysis). An increase in the Bristol Stool Form Score was associated with increasing doses of RSB at visit 5 (*p* = 0.046 for the ITT analysis and 0.0038 for the PP analysis).

### Temporal effects observed across dosing groups

Over the course of the 6 week study, all groups saw improved GI symptoms with decreases in the average scores of PROMIS diarrhea, constipation, and gas and bloating (see Table 2). Significant decreases over time in all groups were observed at 2, 4, and 6 weeks after supplement initiation for the PROMIS T-score of gas and bloating. The Bristol Stool Form score means remained between 3 (“like a sausage but with cracks on its surface”) and 5 (“soft blobs with clear-cut edges, passed easily”) (54) throughout. In the PP analysis (see Supplementary Table S3), time had an effect on a short-chain fatty acid, with butyrate higher at visit 4 (*p* = 0.03). The average sleep disturbance score was lower at each subsequent visit for all groups consuming any quantity of RSB in both ITT and PP analyses.

### Fiber and RSB consumption

The fiber intake from the ASA24 dietary recall data was not identified as a significant covariate in any analyses. Table 3 shows the dietary intake averages and standard deviations, which were reported in between visits. Table 4 shows the RSB consumption as determined by self-reported product dosing logs, product weight calculations, and smart cap usage. These three methods of RSB consumption estimates were used in calculating the dose effect of RSB for the reported outcomes and the significant findings are presented in section The effect of the dose of the blend as calculated by smart cap usage.

**Table 3:** Fiber intake per day, in grams, from self-reported ASA24 results, ITT analysis.

**ASA24**	**PS**	**RSB111**	**RSB122**	**RSB123**
1	16.38 ± 15.58	14.96 ± 7.35	21.57 ± 15.38	15.74 ± 12.46
2	20.20 ± 14.83	19.66 ± 14.7	18.62 ± 11.75	17.90 ± 9.96
3	22.81 ± 10.14	20.48 ± 8.34	24.89 ± 21.05	20.60 ± 12.96
4	19.06 ± 9.70	19.85 ± 12.14	20.37 ± 11.69	22.56 ± 14.56
5	23.29 ± 17.86	16.63 ± 6.30	21.56 ± 12.39	15.92 ± 8.90

Data are means ± standard deviation (SD).

**Table 4:** RSB intake presented as a percentage compliance to protocol-directed doses, ITT analysis.

	**Dosing log**				**Product weight**				**Smart cap usage**			
**Visit**	**PS**	**RSB111**	**RSB122**	**RSB123**	**PS**	**RSB111**	**RSB122**	**RSB123**	**PS**	**RSB111**	**RSB122**	**RSB123**
3	98 ± 5	98 ± 5	94 ± 10	90 ± 10	88 ± 14	92 ± 24	88 ± 15	86 ± 28	75 ± 27	78 ± 32	56 ± 23	67 ± 13
4	98 ± 3	92 ± 15	96 ± 5	95 ± 10	80 ± 11	83 ± 22	91 ± 15	89 ± 24	37 ± 41	59 ± 46	47 ± 27	42 ± 27
5	94 ± 9	93 ± 14	87 ± 33	88 ± 31	89 ± 12	89 ± 16	98 ± 37	87 ± 34	69 ± 51	58 ± 38	61 ± 36	36 ± 26

Data are means ± standard deviation (SD).

### The effect of the dose of the blend as calculated by smart cap usage

Using the dose of RSB as calculated by smart cap openings (see Methods), associations of RSB dose with decreases in diarrhea was consistent with the ITT and PP analyses (*p* = 0.017). Using the cumulative dose over time, as determined by smart cap openings or the RSB weight or dosing log, a decrease in constipation and the Bristol Stool Form number at visit 5 were associated with increased intake of RSB (all *p* < 0.05).

**Figure 2:**
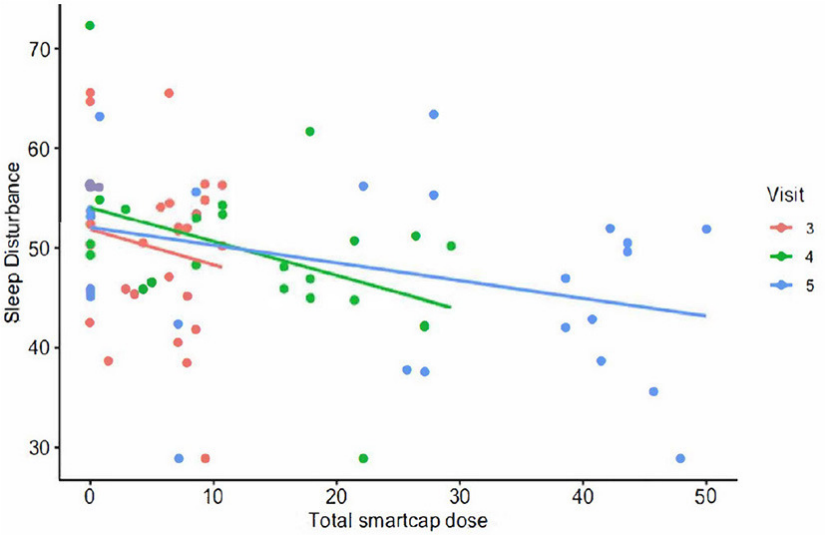
The PROMIS T-score for sleep disturbance plotted vs. total smart cap dose of RSB.

Lower sleep disturbance score was associated with increasing cumulative doses of RSB as calculated by smart cap openings by V4 (4 weeks) with *p* = 0.04 (see Figure 2). The change in T-score corresponded to a mean decrease of 2.23 in sleep disturbance after 2 weeks.

The magnitude of effect using 10 g of total smart cap dose was an increase in total SCFA of 9.60 mmol/g after 4 weeks. After 2 weeks the magnitude of effect consumption of 10 g of total smart cap dose corresponded to the following T-score changes: a 3.60-unit decrease in diarrhea, 4.63-unit decrease in gas and bloating, and a 2.36-unit decrease in constipation score after 2 weeks.

### Safety and tolerability

No severe adverse events occurred. Most participants tolerated the supplement. Per person, the number of adverse events (AEs) possibly or probably related to the potato starch was 0.6, while those related to RSB was 0.5. These AEs included bloating, constipation, fatigue, flatus, cramping, diarrhea, or reflux symptoms. The participant who discontinued the study due to insomnia due to fecal urgency was in the PS group, and the physician determined that 75% of the adverse event was resolved upon discontinuation 2 weeks later. The participant who discontinued after one dose of 30 g of RSB due to severe bloating and constipation that was possibly related to RSB fully recovered from these symptoms prior to the follow-up visit 2 weeks later.

### Exploratory microbiome findings

A permutational multivariate analysis of variance (PERMANOVA) of the pairwise distances [using unweighted, weighted UniFrac distance, and Robust Aitchison PCA (RPCA)] determined that there were no significant differences in the overall microbial composition based on batch (extraction plate, sequencing run), age, race, sex, alcohol consumption (as identified by the selected “track”), diet (calories, carbs, fat, protein, or fiber), or the supplement group at baseline or at the last visit, V5 (see Supplementary Figure S3). Longitudinal analyses of beta diversity across all timepoints also did not reveal significant shifts in the overall microbial composition (see Supplementary Figure S4). Moreover, linear mixed effects models incorporating RSB group did not explain the variance significantly in alpha diversity metrics.

**Figure 3:**
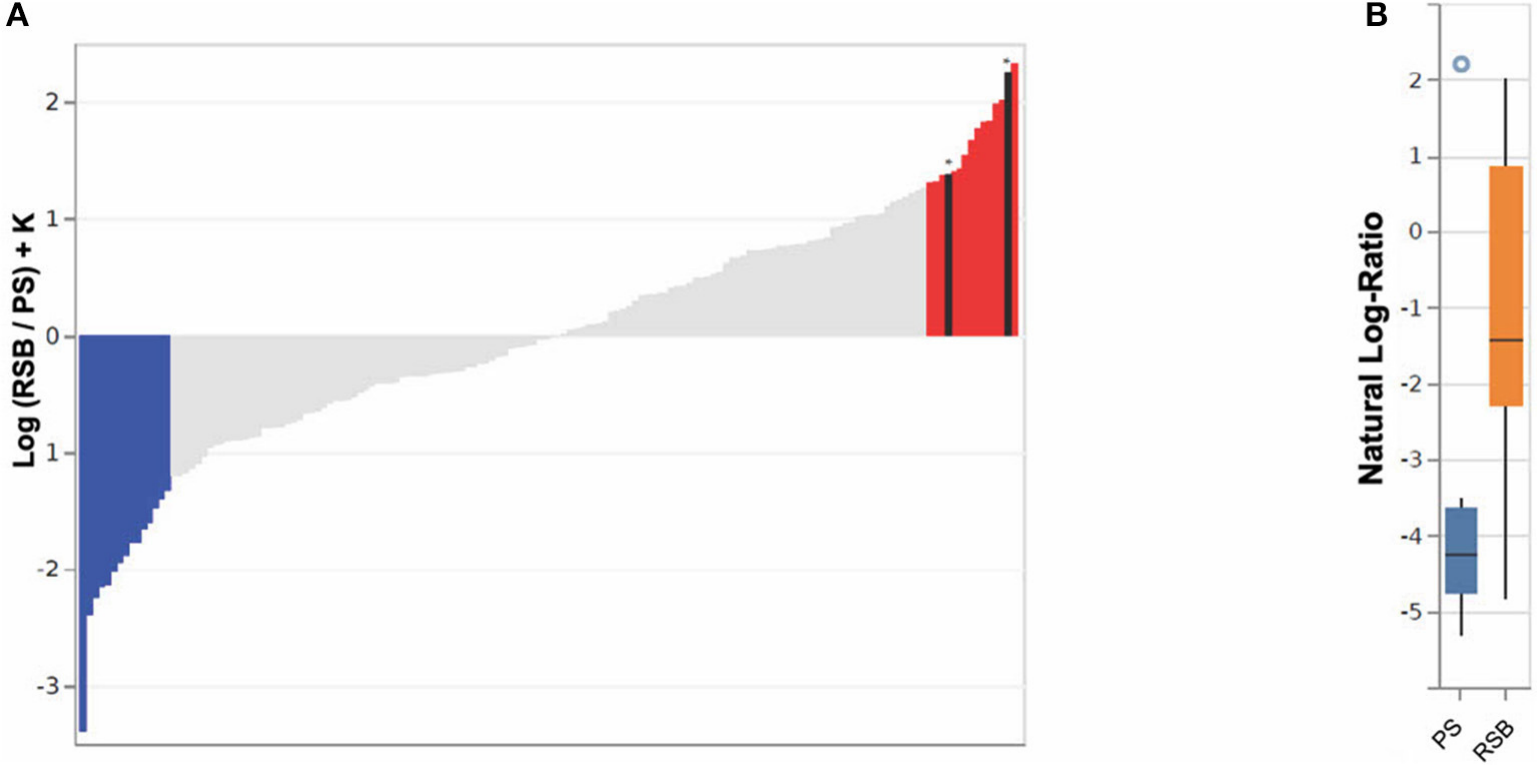
Bacterial ASVs associated with RSB or PS consumption at time point five, using 16S analysis. **(A)** A rank plot highlights the differentials for the top 10% of ASVs (*n* = 15) associated with RSB (colored in red) and the bottom 10% (*n* = 15) associated with PS (colored in blue). ASVs classified as *Akkermansia muciniphila* (second from right) and *Faecalibacterium prausnitzii* (twelfth from right), (see Table S4) are shown in black with an asterisk above. The y-axis shows the log-fold change known up to a bias constant K. **(B)** Boxplot of the log-ratio of the two ASVs assigned as *Akkermansia muciniphila* and *Faecalibacterium prausnitzii* over the top 10% (*n* = 15) associated with PS.

The impact of RSB was further evaluated through Songbird differential abundance analysis at the last time point, Visit Five. This type of analysis allows for a more robust evaluation of differential abundance, accounting for the compositional nature of microbiome data by expressing abundance as log-ratios rather than simple relative abundance measures. The Songbird model utilizing information on whether a participant received any RSB resulted in a Q2 score of 0.02, indicating the model is more predictive of microbial composition when including RSB supplementation as a covariate than the null model. Meanwhile a model utilizing information on RSB amount (specific RSB and PS groups) resulted in a Q2 score of −0.02, suggesting low predictive value. Therefore, analysis of model output was continued only for all RSB groups combined compared to the PS group. The ASVs were ranked in order of association with RSB vs. PS based on a log-fold change in abundance between groups (see Figure 3A) (51). The log-ratios of the top vs. bottom 10% of ASVs associated with RSB were found to be significantly different between the RSB and PS groups at the last visit [Kruskal-Wallis H(1) = 4.69, *p* = 0.030], while log-ratios of these same taxa were not significantly different between the RSB and PS groups at baseline [Kruskal-Wallis H(1) = 2.43, *p*-value = 0.12].

The ASVs most associated with RSB or PS, along with their assigned taxonomies, for Figure 3A are listed in Supplementary Table S4. ASVs classified as the species *Akkermansia muciniphila, F. prausnitzii*, and *Alistipes onderdonkii* were among those most associated with RSB consumption. ASVs assigned as belonging to the families *Verrucomicrobiaceae, Rikenellaceae, Ruminococcaceae, Lachnospiraceae, Christensenellaceae, Bacteroidaceae*, and *Mogibacteriaceae* were also identified in the top 10% ASVs associated with RSB. ASVs included in *Mogibacteriaceae, Blautia, Lachnospira*, and *Bacteroides* associated with both RSB and PS (i.e., appear in both lists). ASVs within *Oscillospira, Coprococcus, Anaerostipes*, and *Bifidobacterium adolescentis* associated with PS. It should be noted that while taxonomic classifications from 16S sequence data may only be reliable to the genus level, it is highly likely that *Akkermansia muciniphila* and *F. prausnitzii* are correctly identified, due to the low diversity of species described in both of those genera. Log ratios of these two ASVs over the top 10% of ASVs associated with PS were found to be significantly larger in individuals who received RSB (−1.26 ± 2.05) vs. those in the PS group (−3.46 ± 2.39) [Kruskal-Wallis H(1) = 4.70, *p* = 0.030] in Figure 3B. At baseline, these ASVs did not have different log-ratios in the RSB vs PS [Kruskal-Wallis H(1) = 0.28, *p* = 0.60].

## Discussion

This trial evaluated increasing doses of RSB, a novel resistant starch blend of MSPrebiotic^®^ potato starch, green banana flour and apple fiber powder, and included a different potato starch of similar resistant starch content as “0 RSB.” In this study we showed that, consistent with previous studies, intake of RS, regardless of source, can provide improvement of GI symptoms. However, we also show that increasing the dose of the proprietary blend, which also included a non-resistant starch source of apple pectin, resulted in significant benefits beyond the benefits obtained from the potato starch in the analytical model. The use of the smart cap monitoring device was essential in finding that increasing doses of RSB improved the sleep disturbance score. Additionally, the unique combination RSB significantly differed from the PS at the end of the consumption period in its associations with ASVs assigned to microbes linked to health.

This trial did not show that increasing doses of the RSB over the course of 6 weeks had a significant impact on SCFA, the primary outcome. By itself, the 10 g/day of the potato starch, which also contained resistant starch, did not show statistically significant shifts in SCFA over time. Possible reasons that we did not observe significant SCFA increases as compared to other studies include and are not limited to elevated baseline levels of SCFA, insufficient quantities of resistant starch or duration of consumption, variability in the diet, variability in participant self-collection, and differences between the test method used and those reported in the literature. The test kit (used for SCFA measurement in this study) from Genova Diagnostics is utilized by functional medicine practitioners and has been documented in studies on patients (55, 56), a probiotic tolerability study (57), as well as a randomized, controlled pilot trial (58). The latter trial did not see a statistically significant change in SCFA after a prebiotic intervention. The average total SCFA ranged from 47–51 micromol/g in the groups, while Genova's internal standard based on their population studies consider ≥23.3 micromol Total SCFA/g as “good.” Similarly, this trial's butyrate levels started in Genova's green healthy zone, above 3.6 micromol/g (averages from 10–12 micromol/g). Thus, the participants did not need to increase butyrate in their feces according to Genova's standards. Additionally, in a study using 22 grams of RS/day over 4 weeks (59) (as compared to 5.5 g/day for 4 weeks in the PS group of this study) butyrate levels did increase for most participants, yet often decreased when baseline levels of butyrate was high, which may have occurred here as well. Although a recent review on resistant starch type II stated that an increase in SCFA is a consistent result (60), the studies reviewed used a higher dose of resistant starch and a different test kit and collection method. Specifically, they did not use the Genova Diagnostics' preservation fluid test kit and self-collection method, and these studies also contained a daily dose of at least 20 grams of resistant starch (compared to 5.5−16.5 g resistant starch in RSB or 6 g resistant starch from the PS in this trial). The previous trial reported on the MSPrebiotic^®^ potato starch in the RSB resulted in significant increases in butyrate using 21 g of resistant starch (30 g raw potato starch)/day for 12 weeks in an elderly population, which shows dose, duration, host age and initial microbiome differences from our trial (28).

While fiber has been shown to be effective in addressing constipation and global IBS symptoms, systematic review results indicate that the benefits are marginal, and insoluble fibers may sometimes aggravate outcomes (61). Both the PS and RSB contained primarily soluble fiber, with similar levels of soluble and insoluble fiber, and both ameliorated constipation, gas and bloating, and diarrhea. We observed small effect sizes for 10 g/day of RSB (corresponding to about 5.5 g/day of resistant starch), and that increasing doses of RSB was associated with more benefit for diarrhea. This suggests that the estimated intake of resistant starch 3–9 g in the typical diets observed in the US, UK, and Australia (62–64) may need to be increased to obtain more benefits as well.

The two ingredients of green banana and apple pectin that make the RSB different from the PS group may be partially responsible for the result that increasing doses of RSB led to improvement in the diarrhea score. Studies in children with persistent diarrhea showed that green banana and apple pectin significantly reduced the duration of diarrhea, vomiting incidents, stool amount, amount of oral rehydration and intravenous fluids, as well as an improvement in intestinal permeability (65, 66).

Although there were no significant findings for wellbeing and sleep in the ITT analysis, the cumulative smart cap dose used for RSB consumption showed a significant association on sleep disturbance score in addition to the benefit to decreasing diarrhea. Sleep disruption is often reported in IBS and has been shown to predict next-day symptoms in women with IBS (67). Thus, in addition to resolution of GI disturbances, improving sleep is a logical target, as it behaves as a potential cause or leading indicator of GI symptoms. It is interesting to note that the cumulative dose of RSB as calculated by smart cap usage was associated with a reduction in sleep disturbance at the end of four weeks. Taken together with the observation that an ASV classified as *Bacteroides* was associated with RSB, a possible mechanism for sleep benefit is that the GABA production by *Bacteroides* members (68) may help with sleep. However, a separate ASV also classified as *Bacteroides* was also associated with PS, highlighting a need to better understand the potential role of specific species or strains of bacteria on such outcomes. Future studies with more smart cap or monitoring data as well as greater understanding the microbiome and metabolites and rigorous sleep monitoring via appropriate devices could further elucidate the actual impact on sleep.

The exploratory microbiome findings suggest RSB consumption is associated with ASVs that are beneficial to GI health. The association with an ASV classified as *F. prausnitzii* showed clinical support for results of *in vitro* studies of apple pectin supporting the growth of *F. prausnitzii* strains. Although the SCFA did not appear to be changed by RSB intake as compared to the PS intake, RSB consumption was associated with butyrate-producer *Faecalibacterium* and acetate-producer *Akkermansia*. The review on resistant starch II consumption using more than 20 g of resistant starch per day in most studies showed that *Ruminococcus bromii* and *F. prausnitzii* were associated with resistant starch intake, which is consistent with the finding reported here for RSB intake while the *Bifidobacterium adolescentis*, also previously reported to associated with resistant starch intake, associated with the PS which also had resistant starch (Supplementary Table S4) (62). More research may need to be done to determine which of the bacteria prefer different types of resistant starch. According to a review of dietary fibers, Bacteroides is among the microbial genera where some species can ferment pectin (69), which supports the finding of *Bacteroides* association with RSB (containing the apple pectin) in this study. Finally, certain proportions of *Clostridiales* such as *Oscillospira* and *Lachnospiracea* were reduced frequently in other studies (62) and this trial showed association of the abundance of those members with intake from the PS or RSB which both contained RS.

Higher levels of *Akkermansia* are also found in healthy individuals' feces than in feces from individuals with inflammatory or metabolic diseases (70). The families to which ASVs associated with RSB are assigned, *Verrucomicrobiaceae, Rikenellaceae, Ruminococcaceae, Lachnospiraceae, Christensenellaceae*, and *Mogibacteriaceae*, were also enriched in long-lived families in a recent study (71). Additionally, the family Christensenellaceae consistently has been associated with reduced visceral fat mass and leanness (72), and *Christenellaceae, Mogibacteriaceae*, and *Rikenellaceae* were associated with leanness, longevity, healthy aging, and protective of cardiovascular and metabolic disorders (73, 74).

### Limitations

Major limitations included the heterogeneity and health of participants in the study's recruitment cohort, the circumstances of the COVID-19 pandemic-associated lockdown, significant participant dependence for self-reported measures and stool collection, sample size limitations, and missing samples. The small sample size, study design (RSB v. Emsland-sourced raw potato starch), and different microbiome analytical methods preclude comparison to prior research using MSPrebiotic^®^ raw potato starch (RPS) (27, 28, 75, 76) or further subgroup analysis with baseline microbiome and smart cap data.

Heterogeneity of health status may have precluded differences in SCFA results as well. In a systematic review and meta-analysis of fecal SCFAs in patients with irritable bowel syndrome (IBS), those with constipation-dominant IBS had lower propionate and butyrate, while those with diarrhea-predominant IBS had higher fecal butyrate compared to healthy controls' fecal SCFAs (77).

The good health of the participants did not allow for much improvement in several outcomes, as can be observed by baseline scores. The study recruited for generally healthy people with at least one GI symptom of constipation, irregularity, or bloating; individuals with diarrhea were not specifically recruited, and specific Rome criteria or other standards were not used to characterize the population relative to specific subtypes of IBS. Some if not all participants would likely fall into the “healthy” description of controls in case-control studies observing differences with IBS (78). The range of “healthy” stool frequency is generally accepted to be between three bowel movements per day and three bowel movements per week (78), which is rather a wide window and fully encompasses the study cohort's stool frequency throughout the study.

Despite using random number assignments, there was a distribution of more women in groups consuming the blend which was an unfortunate limitation. Available sample size does not allow for subgroup analysis to identify the impact of this distribution.

Finally, the original choice for a control was a potato starch that had prior test results showing little to no RS; however, *post-hoc* test results showed that the potato starch had similar RS content to the RSB, which resulted in a study design lacking a true control without RS. However, it did allow for an evaluation of whether a multi-source RS blend delivered different results than a single source potato starch and whether different dosage effects could be detected. We anticipate that a follow-up study would further show that RSB results in even more dramatic improvement in GI symptoms and sleep, and more significant shifts in gut microbiota over using a supplement free of RS.

## Conclusions

This study suggested that a novel resistant starch blend exhibited benefits beyond those of a standard resistant starch, which also improved GI symptoms. The novel RSB improved GI symptoms, particularly diarrhea, in 2 to 6 weeks with 10, 20, or 30 g doses tested. With the smart cap, this study confirmed expected outcomes such as increasing Bristol Stool Form, decreasing diarrhea and constipation scores, and showed that the cumulative RSB dose led to a decrease in the sleep disturbance after 4 weeks.

The exploratory microbiome analysis showed that a novel RSB is associated with *Akkermansia muciniphila, F. prausnitzii*, and other ASVs belonging to families of bacteria that have previously been associated with longevity and health.

For the field of resistant starch research, the trial suggests that a supplementary dose of 5–6 g of resistant starch consumed above normal dietary intake (estimated at 3–9 grams) per day, at least in the course of 2 to 6 weeks, is insufficient to move SCFA levels. While it may not be necessary to consume 20 or more grams of supplemental RS for four or more weeks as has been showed in previously reviewed studies to obtain SCFA increases, it is still unknown what a “good” level of fecal SCFA is. At the same time, this trial also points to the difficulties in resistant starch testing, which varied in its results and has been the subject of debate (Englyst or other methods).

This original research provokes the field to consider unique combinations of prebiotics and resistant starches rather than single sources while at the same time calling for more rigorous and standardized methods including adherence monitoring to evaluate the impact of dietary interventions on the GI.

Our multifaceted monitoring of supplement consumption indicated significant variation in the amount of the supplement consumed, even within the same dosing group. As a result, future research may require larger participant samples in order to account for differences in compliance. Since the smart cap monitoring suggested an impact on sleep disturbance that was otherwise not apparent, future studies in this area would benefit from this or similar technology. Future studies with enhanced control over the population, stricter protocol requirements, and larger sample sizes to account for lack of adherence to protocol-directed study product consumption may provide future insight into personalizing novel blends of prebiotics, RS, and fibers at appropriate doses to modulate the microbiome and promote health.

## Data availability statement

The datasets presented in this study can be found in online repositories. The names of the repository/repositories and accession number (s) can be found at: https://www.ebi.ac.uk/ena, ERP134986; https://qiita.ucsd.edu, 13692.

## Ethics statement

The studies involving human participants were reviewed and approved by Aspire Institutional Review Board. The patients/participants provided their written informed consent to participate in this study.

## Author contributions

Conceptualization, validation, writing—original draft preparation, and funding acquisition: NP. Data curation: NP, PLMC staff, and CMI. Methodology, formal analysis, and visualization: NP, DH, and BN. Software: DH and BN. Supervision of PLMC staff and investigation: JL. Resources: all authors. Writing—review and editing: NP, DH, BN, and SS. Supervision: NP, SS, IL, RK, and DM. Project administration: NP, SS, and IL. All authors have read and agreed to the published version of the manuscript.

## Funding

This research was funded by Metagenics, Inc.

## Conflict of interest

Authors IL and NP were previously employed through data analysis and initial drafting of the manuscript by Metagenics, Inc., the provider of the resistant starch blend and sponsor of the entire research. Author JL is the co-owner of Personalized Medicine, Inc., which is a consultant for Metagenics, Inc. The remaining authors declare that the research was conducted in the absence of any commercial or financial relationships that could be construed as a potential conflict of interest. The authors declare that this study received funding from Metagenics. The funder had the following involvement in the study: design and initial writing of the article.

## Publisher's note

All claims expressed in this article are solely those of the authors and do not necessarily represent those of their affiliated organizations, or those of the publisher, the editors and the reviewers. Any product that may be evaluated in this article, or claim that may be made by its manufacturer, is not guaranteed or endorsed by the publisher.
