# Supplementary material for: The gastrointestinal and microbiome impact of a resistant starch blend from potato, banana, and apple fibers: A randomized clinical trial using smart caps
Source: Front Nutr. 2022 Sep 29;9:987216. doi: 10.3389/fnut.2022.987216 (PMC9559413; doi:10.3389/fnut.2022.987216)
Supplement: Supplementary file 1 [file Presentation_1.zip › Figure S1.pdf]

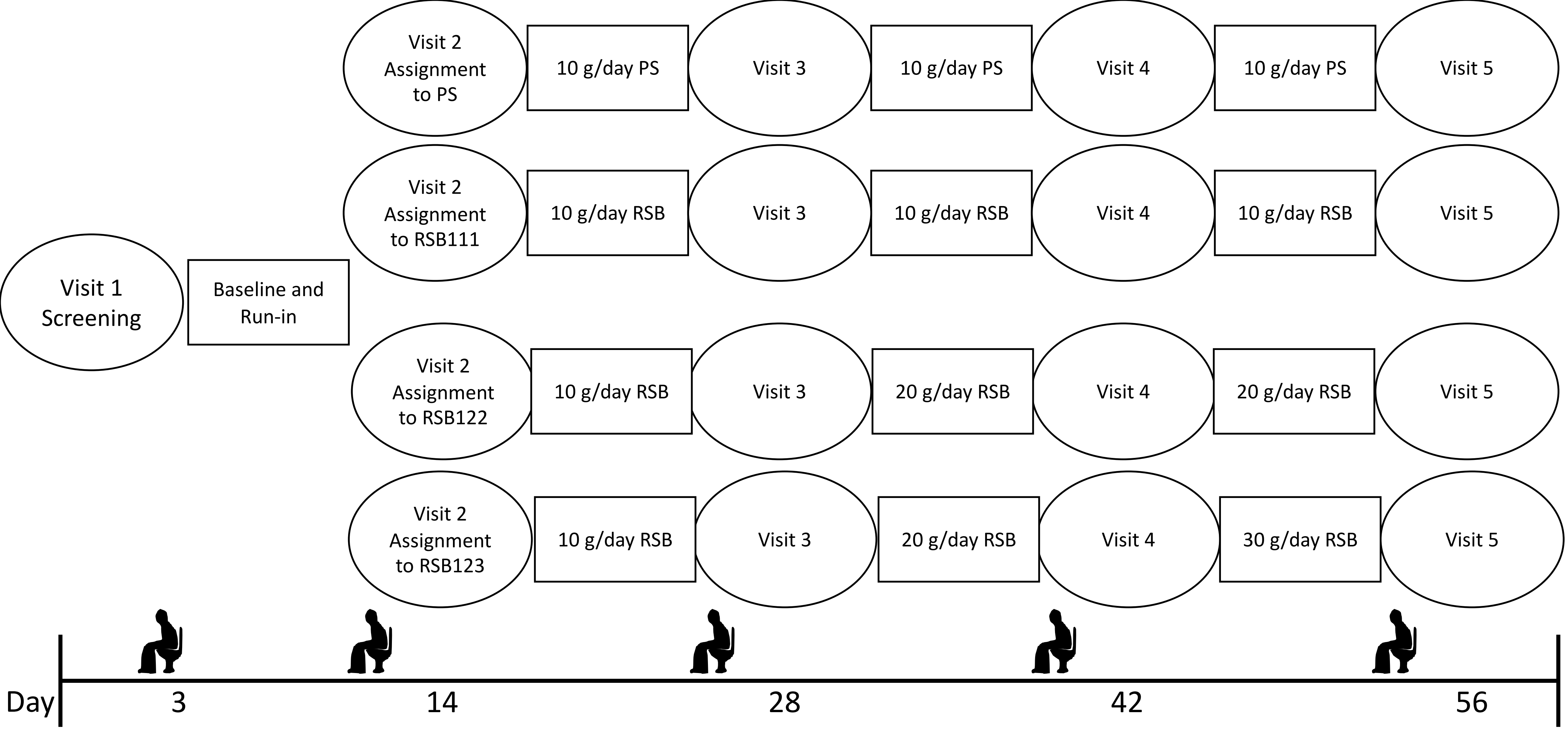

Data collected corresponding to each visit or timepoint:

- Stool samples (Genova kit for SCFA, American Gut for microbiota 16S and metagenomics) within 3 days of visit
- PROMIS questionnaires on GI symptoms, wellbeing and sleep
- ASA24 Food Intake
- Bristol Stool Form rating and day and time of stool production for 7 days
- Product Consumption (Dosing log,
- Vital signs in-person study permitting
- Blood draws at 1, 2 and 5, in-person study permitting

Figure S1. Study Design.
