# Supplementary figures and images for: The gastrointestinal and microbiome impact of a resistant starch blend from potato, banana, and apple fibers: A randomized clinical trial using smart caps
Source: Front Nutr. 2022 Sep 29;9:987216. doi: 10.3389/fnut.2022.987216 (PMC9559413; doi:10.3389/fnut.2022.987216)

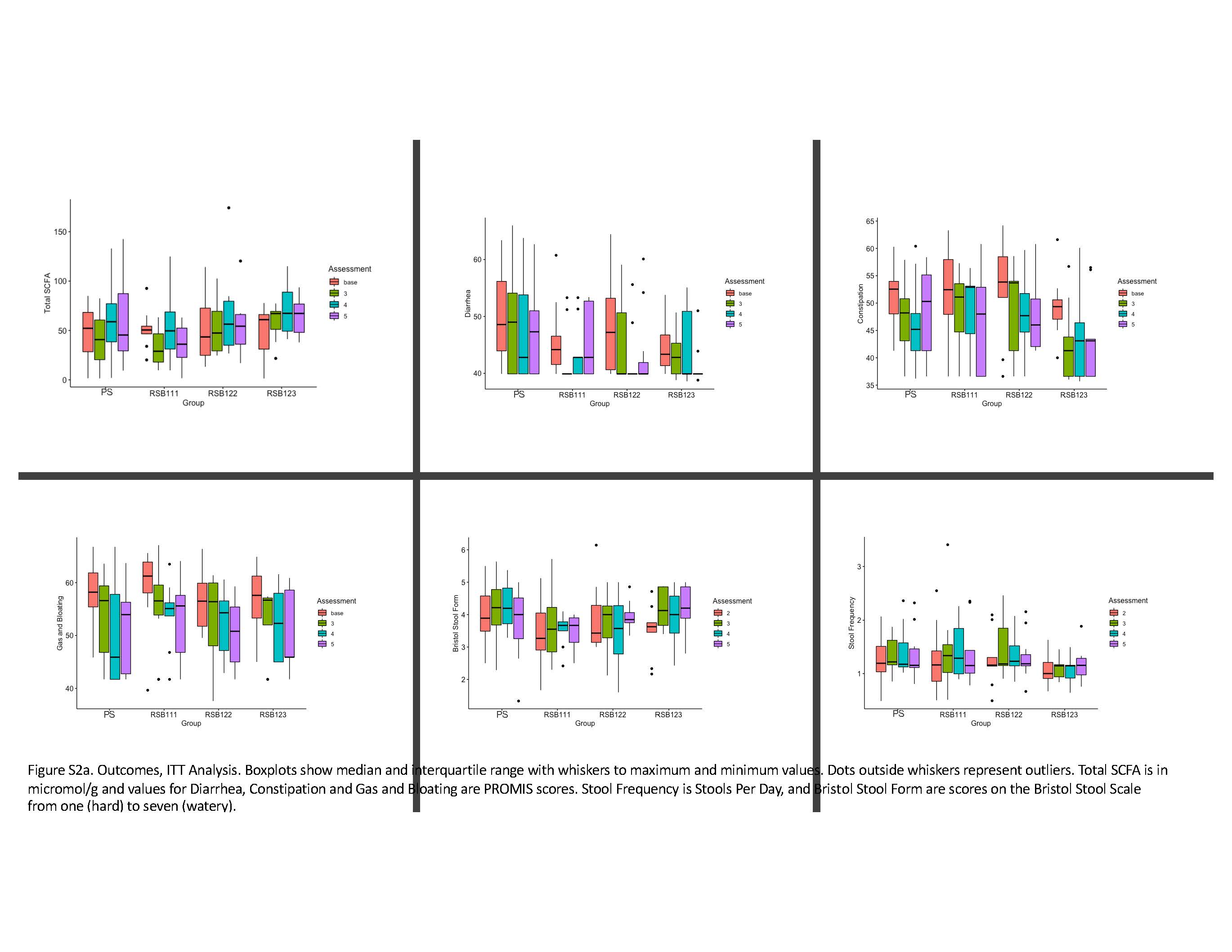

Supplement: Supplementary file 1 [file Presentation_1.zip › Figure S2a.JPEG]

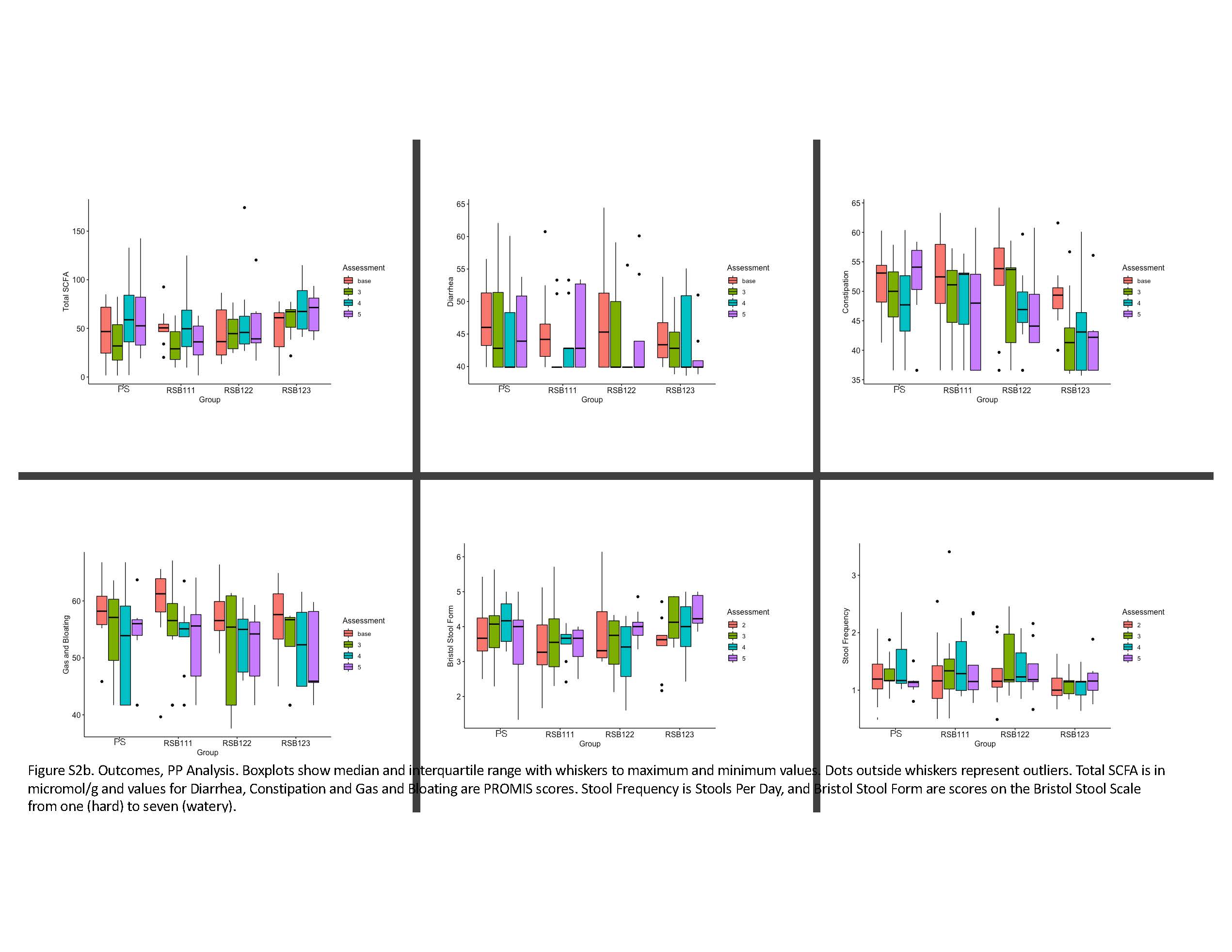

Supplement: Supplementary file 1 [file Presentation_1.zip › Figure S2b.JPEG]

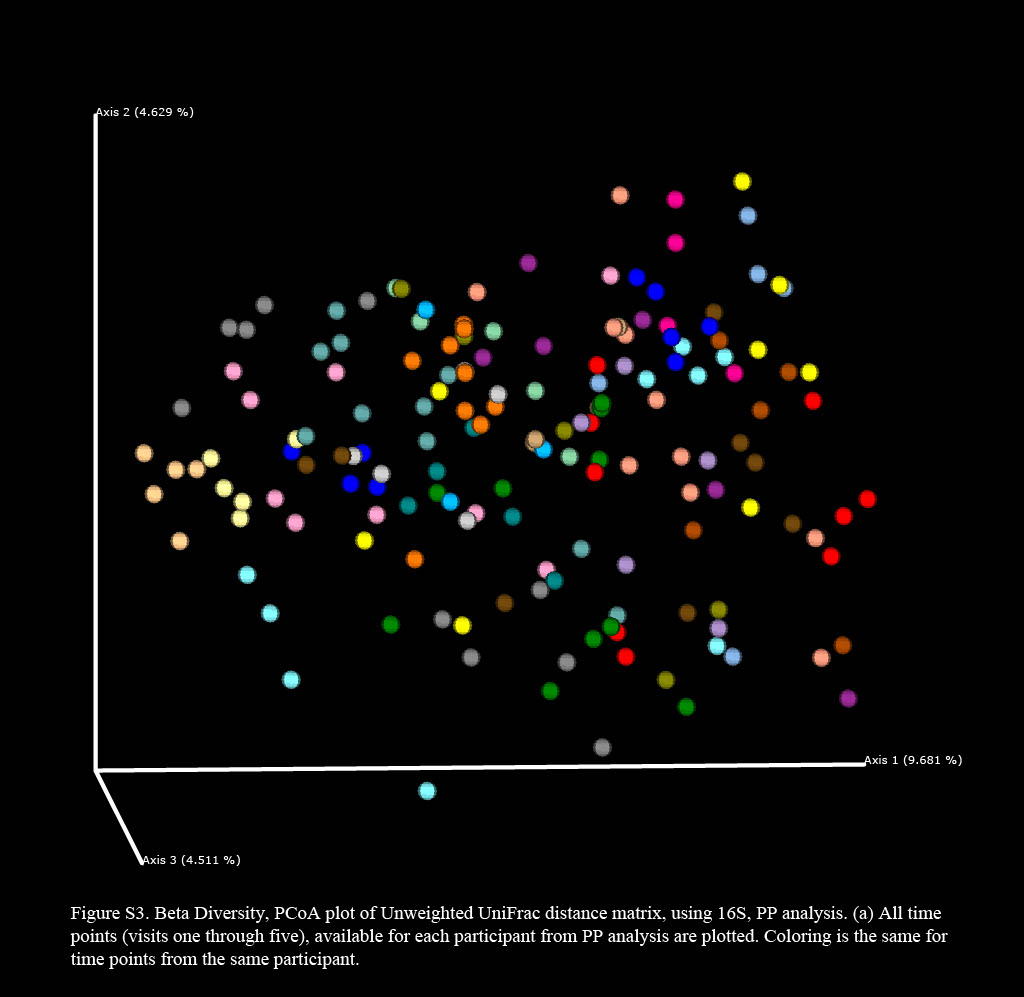

Supplement: Supplementary file 1 [file Presentation_1.zip › Figure S3a.JPEG]

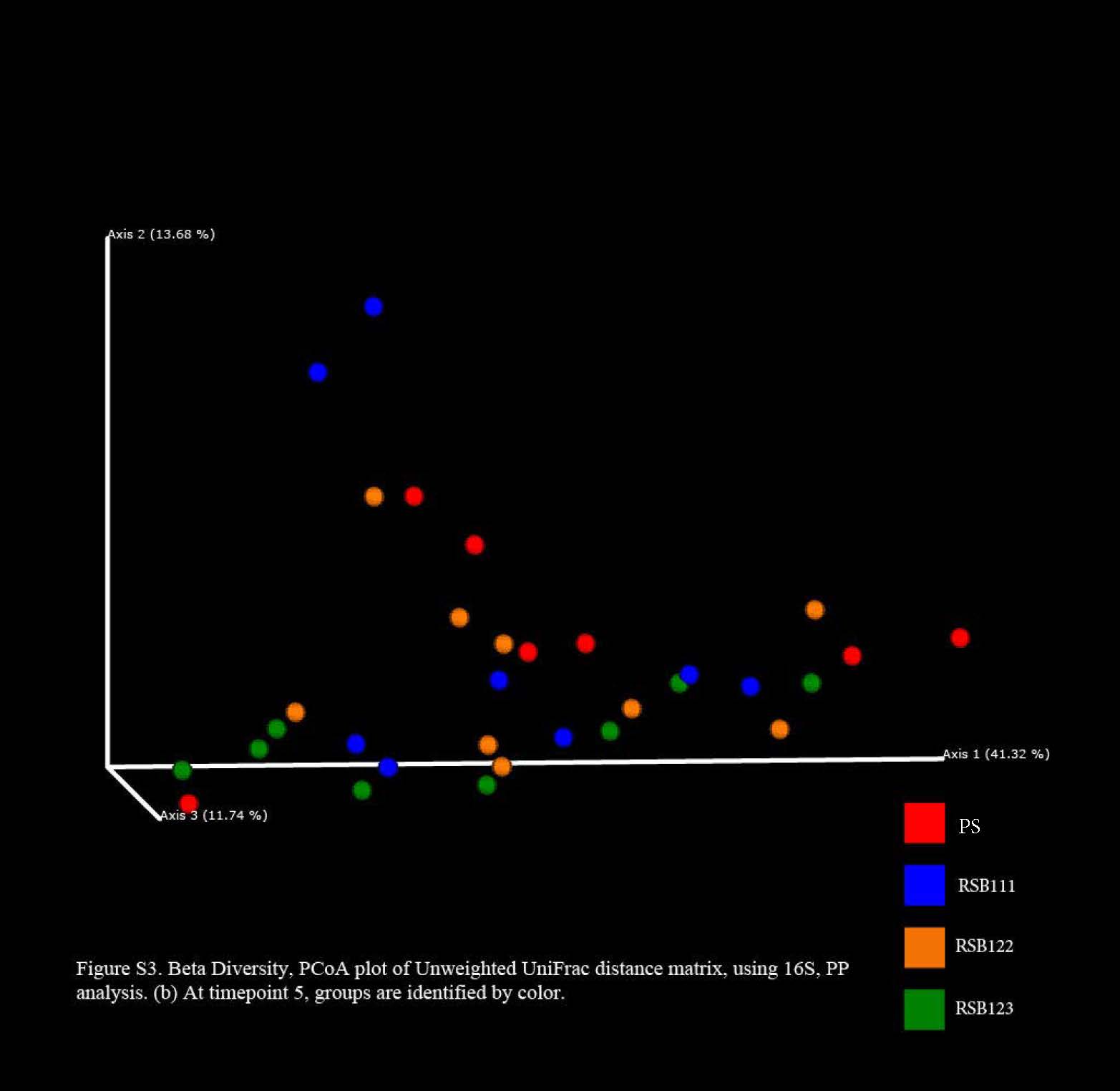

Supplement: Supplementary file 1 [file Presentation_1.zip › Figure S3b.JPEG]

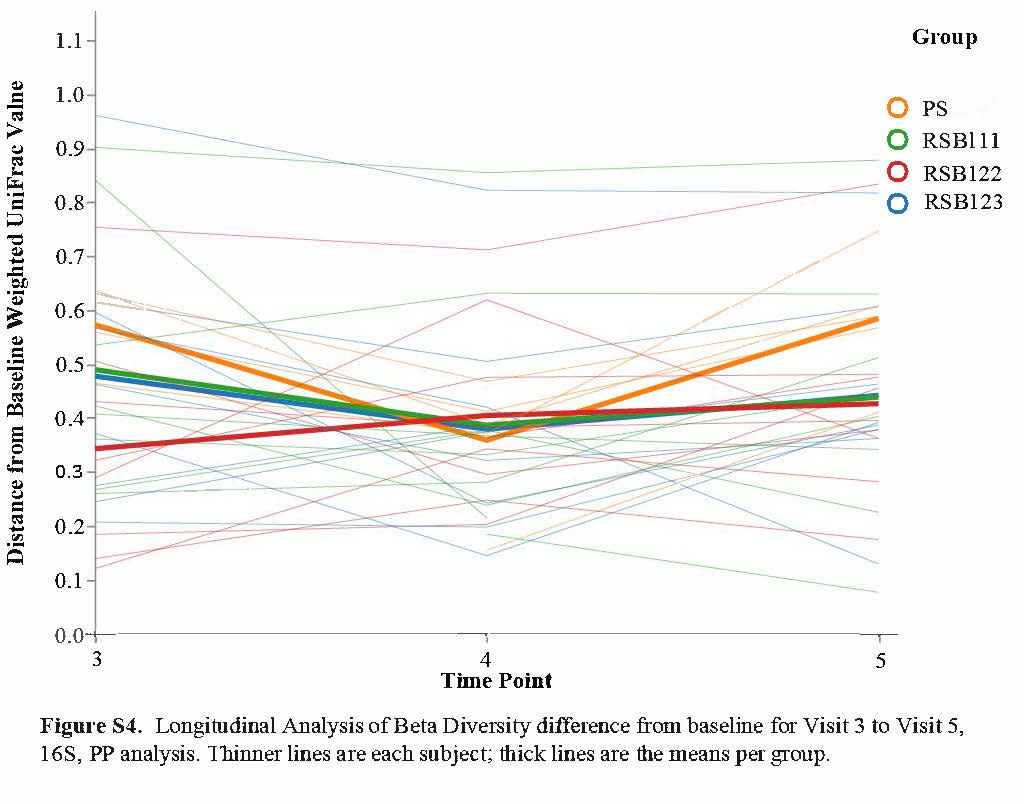

Supplement: Supplementary file 1 [file Presentation_1.zip › Figure S4.JPEG]
